# Persons’ Experiences of Suffering from Nephrotic Syndrome

**DOI:** 10.1111/jorc.12307

**Published:** 2019-11-19

**Authors:** Anneli Jönsson, Thomas Hellmark, Anna Forsberg

**Affiliations:** ^1^ Department of Clinical Sciences, Nephrology, Faculty of Medicine Lund University Lund Sweden; ^2^ Department of Health Science, Faculty of Medicine Lund University Lund Sweden; ^3^ Biomedical Centre Skåne University Hospital Lund Sweden; ^4^ The Thoracic Surgery Lund Skåne University Hospital Lund Sweden

**Keywords:** Ambiguity, Illness, Nephrotic syndrome, Uncertainty, Self‐Management/Self‐Care, Chronic Kidney Disease

## Abstract

**Background:**

Little is known about health and wellbeing among patients with nephrotic syndrome (NS), despite it being a serious condition in patients with renal failure. In order to promote health, it is important that both healthcare professionals and patients are aware of the signs and symptoms of the disease.

**Objectives:**

The aim was to explore patients’ experience of suffering from nephrotic syndrome.

**Design:**

An inductive, qualitative method.

**Participants:**

Ten adult patients with either newly diagnosed or a relapse of NS treated in a University hospital, south of Sweden from February 2016 to February 2019.

**Measurements:**

Data were collected using open‐ended interviews and analysed by means of Lindseth and Norberg's phenomenological‐hermeneutical method.

**Results:**

Suffering from NS meant being a stranger in an unfamiliar world of symptoms, signs and medical treatment without professional guidance or piloting, illustrated by four themes: *Feeling ill and well at the same time, Being passively adherent, Being in uncertainty*, and *Trying to comprehend and cope*.

**Implications for practice:**

The result provides an in‐depth understanding of the illness experience among patients with NS and constitutes a foundation for clinical guidelines on treatment, follow‐up and health promotion.

**Conclusion:**

Patients with NS end up in a state of ambiguity due to a profound knowledge deficit that causes uncertainty and a lack of self‐management. The experienced lack of professional self‐management support is partly compensated for by social support from relatives, enabling those with NS to manage everyday life in a reasonable way.

## INTRODUCTION

Little is known about the experienced health and well‐being of patients with nephrotic syndrome (NS), despite the fact that it is a serious condition. Thus, the rationale behind this study was to understand the patients’ perspective of suffering from NS.

NS is not a disease per se, but a condition characterised by severe proteinuria, hypoalbuminemia, oedema and hyperlipidaemia. A wide range of primary or secondary renal diseases can cause NS. A common cause of NS in adults is membranous nephropathy and in children minimal change nephropathy (Hull & Goldsmith, 2008). Although it is a serious condition, NS is relatively rare with about three new cases per 100,000 diagnosed annually (Hull & Goldsmith, 2008). One of the main complications of NS is venous thrombosis in the deep veins of the lower limbs (Kerlin *et al*., 2012). Other complications are end‐stage kidney disease (ESKD), infections and cardiovascular events (Kodner, 2016). The treatment of NS addresses the cause as well as the symptoms. The most common treatment of the symptoms includes loop diuretics, angiotensin‐converting enzyme inhibitors and/or angiotensin II receptor antagonists, statins and anticoagulants (Hull & Goldsmith, 2008). Other ways of treating the original disease are the use of steroids, Calcineurin Inhibitors and chemotherapy. The outcome of NS is dependent on the underlying causes and proteinuria. Membranous nephropathy and minimal change nephropathy have a relatively favourable prognosis. Without treatment, one‐third of patients with membranous nephropathy achieve spontaneous remission, one‐third develop persistent proteinuria and one‐third progress to ESKD (Cattran & Bernchley, 2017).

### Literature review

Most studies on health‐related quality of life (HRQoL) in patients with a renal disease focus on patients in dialysis or in paediatric care. Studies focusing on HRQoL of adult patients with nephrotic syndrome indicate that the patients have low HRQoL (Libório *et al*., 2012; Shutto *et al*., 2013). A survey with SF‐36 showed that patients with NS have a low HRQoL regarding general health and social functioning compared to the general population (Shutto *et al*., 2013). The fact that patients with NS have worse HRQoL than healthy subjects was also confirmed by Libório *et al*. (2012). In their study patients with NS reported low scores in almost all items on SF‐36 and they also reported symptoms of depression, comparable to those in haemodialysis (Libório *et al*., 2012). Finally, patients with NS have symptoms related to proteinuria such as severe oedema and loss of kidney function, which can cause a reduction in physical or mental health and subsequently the quality of life. In addition, treatment‐related side effects may be associated with poor quality of life. However, a study has shown that patients with NS without treatment also had low HRQoL scores (Gipson *et al*., 2011).

Presumably, NS affects the patients’ everyday life and it raises questions in relation to the disease. However, knowledge about their illness experience is lacking, thus the aim of this study was to explore patients’ experience of suffering from NS.

## PATIENTS AND METHODS

### APPROACH AND DESIGN

This study is inductive and employs a qualitative method. To capture the meaning of the patients’ lived experience of suffering from NS, an approach based on Ricoeur's hermeneutic phenomenology (1976, 1994) was chosen. The choice of Ricoeur's version of interpretive phenomenology was motivated by its focus on the capable human being (i.e. homo capax) and its relationship to personhood and person‐centeredness.

### PARTICIPANTS

Newly diagnosed as well as relapsed adult patients (>18 years old) who became ill with NS and were treated at Skåne University Hospital, Lund between February 2016 and February 2019 were consecutively invited to participate in the study. The inclusion criteria were NS (albumin/creatinine ratio >300–350 mg/mmol), hypoalbuminaemia (<28 g/L) and able to speak and understand Swedish. All participants had been urgently admitted to the nephrology clinic due to an acute relapse or being diagnosed with NS for the first time. At the time of the interview, they had been hospitalised only for a maximum period of five days. Their treatment was either performed in a hospital (n = 5) or at the outpatient clinic (n = 5) enabling them to be at home during night time. During the study period, there were 10 eligible patients (six men and four women, mean age 57.8 years, median 66.5 years, range 21–90 years), all of whom agreed to participate. The diagnoses that caused NS were membranous nephropathy (n* = *7) and minimal change nephropathy (n* = *3) (Table 1).

**Table 1:** Participant characteristics (n = 10)

Age (years)	Gender	Diagnosis
21	M	Minimal change nephropathy
30	F	Minimal change nephropathy
31	M	Membranous nephropathy
36	F	Minimal change nephropathy
65	F	Membranous nephropathy
68	M	Membranous nephropathy
71	M	Membranous nephropathy
82	M	Membranous nephropathy
84	M	Membranous nephropathy
90	F	Membranous nephropathy

### DATA COLLECTION

Data were collected through open‐ended interviews. An interview guide was developed with three opening questions: “Can you please tell me what happened when you became nephrotic or ill?” “Can you please tell me how your health has changed?” and “How did you discover that you were ill?” Probing questions were asked when necessary to generate narratives of the experience. The interview guide stemmed from the aim of the study as well as a long‐standing clinical experience and the interviews lasted for a median time of 33 minutes (range 21–52 minutes). The participants were allowed to choose time and place for the interview and all chose either the ward (n = 5) when they were hospitalised or the outpatient clinic (n = 5) when they were attending for treatment/during follow‐up visits. The interviews, which were audio‐recorded and transcribed verbatim, were performed by one of the authors (AJ).

### DATA ANALYSIS

Lindseth and Norberg's phenomenological hermeneutical method was used to interpret the interviews (Lindseth & Norberg, 2004). This method involves three dialectically interrelated steps comprising naïve reading, structural analysis and comprehensive understanding.

In the first step, the transcribed text was read several times to grasp the meaning. The purpose was for the researchers to become familiar with the content and gain an understanding of the phenomenon as a whole. In the structural analysis, the text was broken down into meaning units. Similarities and differences were identified and condensed into themes and sub‐themes. During the analysis, the themes and sub‐themes were reflected in relation to the naïve understanding in order to validate it. Examples of the structural analysis are presented in Table 2. In the third phase, comprehensive understanding, the themes were reflected on in relation to the research question. The researchers’ preunderstanding and experience of patients with NS were important during the interpretation.

**Table 2:** When analysing the interview texts Lindseth and Norberg's phenomenological hermeneutical method was used (Lindseth & Norberg, 2004). This is an example of structural analysis in the third theme “Being in uncertainty.”

Meaning unit	Condensation	Sub‐themes	Theme
“I don't know what to do. They haven't really explained that to me. If it gets worse I don't really know what will happen”.	“Lack of information about the future”	Doubting the future	Being in uncertainty
“Have I exercised too hard or too much … have I lived too unhealthily”?	“Considered whether one's lifestyle triggered the disease”	Questioning one's own role	

### ETHICAL CONSIDERATIONS

Approval was obtained from The Regional Ethics Board, Lund University (Dnr. 2016/505). The study conforms to the principles outlined in the Declaration of Helsinki (World Medical Association, 2018). The participants received verbal and written information about the purpose of the study. They were informed that participation was voluntary and that confidentiality would be ensured. All participants gave their written informed consent.

## FINDINGS

### NAÏVE READING

The naïve understanding revealed that patients with NS are in a state of ambiguity, i.e. the disease and its consequences do not make sense to them and they have no idea what to expect or do. NS is a condition in which the meaning cannot be defined by the patients. They cannot make sense of something that makes no sense which leads to a complete lack of coherence. Thus, the structural analysis constitutes the meaning of being in ambiguity while suffering from NS.

### STRUCTURAL ANALYSIS

The structural analysis resulted in four themes based on 12 sub‐themes (Table 3) and is presented with quotations from the interview text.

**Table 3:** Structural analysis of the meaning of being in ambiguity among ten patients with a nephrotic syndrome involving main themes and sub‐themes

Themes	Feeling ill and well at the same time	Being passively adherent	Being in uncertainty	Trying to comprehend and cope
**Sub**‐themes	Experiencing physical changes	Avoiding asking questions	Doubting the future	Seeking information
	Not noticing or understanding symptoms	Trusting healthcare professionals	Wondering about causes and prognosis	Acknowledging family support
	Being affected by the disease but managing everyday life	Feeling powerless	Questioning one's own role	Willing to make lifestyle changes

### FEELING ILL AND WELL AT THE SAME TIME

Suffering from NS meant feeling ill and well at the same time. The participants experienced physical changes but did not understand why they had these symptoms. They were unable to connect their physical symptoms to renal disease. The experienced physical changes stemmed from fluid retention in the body, leading to shortness of breath and difficulty breathing.

*“I had to take a deep breath and then it is as if there is no more space.”* (Male, 71 years).

The first signs of disease experienced by the participants were a swollen body and weight gain:

“*The first thing I noticed was that my eyes were swollen, the eyelids. Towards the evening I also noticed that my legs and feet were swollen*.” (Female, 65 years).

The participants did not notice or understand their symptoms, despite being informed that they had renal disease. They were simply unable to relate their signs or symptoms to renal disease even when signs of fluid retention were obvious.

“*From what I have understood, this is a disease that develops slowly. I have read about it, but haven't noticed any signs myself*. (Male, 84 years).

The participants were affected by the disease but still managed everyday life fairly well. As the swollen legs did not affect their ability to walk they were able to carry out the activities of everyday life reasonably well.

“*I have not been limited in my everyday life. I keep up the spirit and my social life is working. So there was no such problem” (Male, 84 years)*.

They experienced excessive swelling in their legs and feet, as a result of which they needed larger shoes and had to rest their legs in a raised position in the evening. Even more troublesome was the treatment with high doses of diuretics, which severely restricted their social functioning and freedom to leave their homes.

### BEING PASSIVELY ADHERENT

Being passively adherent meant relying on and following the healthcare professionals’ recommendations and prescriptions without questioning anything from their position of disadvantage. The participants adhered to the advice and prescriptions, even when they did not fully understand them. The participants also exhibited a kind of powerlessness as they were unable to influence the course of the disease or treatment.

A more or less conscious strategy was to avoid asking questions about the disease or treatment. This approach was due to either a lack of interest in the disease or a personality trait, as some of the participants explained that they were not the type of person who asks questions.

“*The doctor knows what is best and I am not interested in diseases*.” (Male, 71 years).

The participants trusted healthcare professionals in an unreflective and obedient manner. Some were adherent regarding all types of advice, adhered to prescriptions and did exactly what they were told.

“*The doctor says I should take them, so I do. I must obey. I question nothing, neither the injection nor the tablet. They must be good, otherwise I would not have received them*.” (Female, 65 years).

The participants felt powerless because they were unable to influence the disease, which led to a feeling of impotency:

“*If she [the nephrologist] told me to take some pills, I simply had to do that. And to simply follow the advices regarding what to do or not. For me as a patient there is nothing else I can do*” (Male, 68 years).

### BEING IN UNCERTAINTY

Being in uncertainty was about not knowing why one has become ill and what the future holds. The uncertainty arose from lack of sufficient information about the disease or how to live with it. They doubted their future as they had received no information about what to expect, thus did not know whether they would become healthy again or if their condition would deteriorate.

“*I don't know what to do. They haven't really explained that to me. If it gets worse I really don't know what will happen*.” (Male, 21 years).

Due to their experience of a complete lack of information and guidance the participants wondered about what caused the disease and their prognosis. It was impossible to know what to expect in the future and some pondered on whether they had caused the disease themselves due to their lifestyle.

“*I don't know why I got it. And I have never been informed why. So I wonder, why and how? Is it possible to heal? Or is that also unknown*? (Female, 65 years).

Some participants questioned their own role and wondered which lifestyle they would adopt in relation to their illness and if they could create routines that would have a positive effect on the disease.

“*I really don't know how to live now … should I rest … should I take it easy? I really don't know much about it*.” (Female, 30 years).

### TRYING TO COMPREHEND AND COPE

Trying to comprehend and cope meant that the participants struggled to understand their illness and handle it in the best possible way. Those who did not actively avoid asking questions searched for information on the Internet, but the information they found was not always accurate.

“*What kind of information is there about the diagnosis? How do I find the right information*?” (Female, 36 years).

Family support was crucial for managing the situation in a constructive way. Relatives provided instrumental support such as childcare and other necessary practical support in acute situations. They also provided emotional support by phoning and asking about emotions and experiences.

“*My family means a lot. I can imagine that it would have been more difficult if you didn't have someone to talk to*.” (Female, 30 years).

The participants were willing to perform necessary lifestyle changes, but received no guidance, leaving them alone in a limbo of trial and error.

“*I am ready to make changes… but I do not really know what to do, because I think I live quite healthily*.” (Male, 31 years).

### COMPREHENSIVE UNDERSTANDING

Suffering from NS meant being a stranger in an unfamiliar world of symptoms, signs and medical treatment without experiencing professional guidance or piloting. No one seemed to pay any attention to their personal narrative or meaning‐making. Nor were they invited into a partnership and caring relationship by a nephrology nurse or physician. They, therefore, ended up at a threefold disadvantage because they (a) were at the bottom of the hierarchical structure of the healthcare institution (b) were suffering from a severe and disabling disease affecting their existence and (c) had a complete knowledge deficit causing a cognitive disadvantage (Kristensson Uggla, 2014). There was a willingness to learn and understand, but the lack of guidance from healthcare professionals regarding causes, prognosis and self‐management activities increased their suffering and prevented health and well‐being. The participants ended up in a state of constant ambiguity and uncertainty in illness, where self‐management became a mission impossible without reliable support. This is coherent with other studies of uncertainty in illness, e.g. diabetes mellitus, Parkinson's disease and heart transplantation (Johnson Wright *et al*., 2009; Almgren *et al*., 2017).

## DISCUSSION

To the best of our knowledge, this is the first qualitative study of patients’ lived experience of being in an acute phase with NS and under immunosuppressive treatment. The findings suggest that being in an acute phase of NS means being in ambiguity involving strong feelings of uncertainty. Previous studies show that patients with advanced illness are in a state of uncertainty, which leads to ambiguity or loss of coherence (Almgren *et al*., 2017; Etkind *et al*., 2017). According to Beanlands *et al*. (2017), patients with NS have difficulty understanding the seriousness of their disease because they experience no or few symptoms. It was confusing not having symptoms that one could associate with renal disease. When the intensity and location of symptoms are inconsistent, it generates uncertainty (Mishel, 1988). Mishel (1988) defines uncertainty in illness as “the inability to determine the importance of illness‐related events” (p. 225), which is consistent with our findings. Despite the uncertainty, there was a willingness to learn and understand in order to practice self‐management. However, the lack of guidance from healthcare professionals only increased the suffering. Self‐management is defined as the activities people undertake to create order, discipline and control in their lives (Kralik *et al*., 2004). The participants could not undertake these activities due to their uncertainty and lack of coherence. Costantini *et al*. (2008) claim that there is a need to provide early disease‐specific information so that the patient can learn to live with chronic renal disease. Self‐management in renal disease involves the development of knowledge, skills and behaviours that are necessary for managing the disease and treatment (Novak *et al*., 2013).

In the Innovative Care for Chronic Conditions (ICCC) framework, the World Health Organization (2002) states that a partnership between healthcare professionals and patients should involve motivating and preparing patients and their families in order to achieve better outcomes in chronically ill individuals. In our study the participants were not invited to a partnership with the healthcare professionals and thus ended up at a threefold disadvantage; institutional, existential and cognitive (Kristensson Uggla, 2014), which can lead to a state of ambiguity, uncertainty and a feeling of impotency. It is essential that healthcare professionals invite patients and relatives to a partnership, as otherwise there is a risk of the patients remaining at a disadvantage and being depowered instead of empowered, which has a negative impact on self‐management. According to Ricoeur (2011), a person is capable and has the ability and willingness to speak, act, tell and take responsibility. In order to promote health and support self‐management, healthcare professionals should strengthen the patient's power as far as possible, starting from her/his wishes and individual situation (The Swedish Patient Act, 2014, p. 82).

Patients with any form of chronic disease have a lower quality of life compared to the general population. In addition, they are also at increased risk of co‐morbidity and mortality (Golics *et al*., 2013). Health and illness are unique to each individual, which affects the symptom experience, management and outcome. Symptom experience includes the individual's perception of how she/he usually feels or behaves and responds to symptoms (Dodd *et al*., 2001). A symptom is also an indication of a disease detectable by the person or by others such as healthcare professionals. An understanding of the illness perspective of patients with NS might not only prevent morbidity and mortality but also contribute to a foundation for person‐centred care.

### METHODOLOGICAL CONSIDERATIONS

All eligible patients were included in the study and the large age distribution among the participants can be viewed as a strength. Irrespective of age, the participants had similar illness experiences. During the interpretation, the researchers reflected on whether their pre‐understanding influenced the comprehensive understanding. Although one of the researchers has extensive experience of working in a renal department, it was offset by the fact that the others did not have such experience. To ensure trustworthiness and prevent the researchers’ pre‐understanding from influencing the result, the researchers AJ and AF analysed the data together. One limitation is that this is a single centre study. Traditions and NS specific treatment at the hospital from which the participants were recruited may have affected their experience. Furthermore, the participants only reflect a Swedish speaking population, thus a multi‐ethnic perspective on the disease is lacking.

## CONCLUSION

Patients treated for acute NS end up in a state of ambiguity involving a profound knowledge deficit, which leads to uncertainty and lack of self‐management. The experienced absence of professional self‐management support is partly compensated offset by relatives’ social support, thus enabling the participants to manage everyday life reasonably well.

### IMPLICATIONS FOR CLINICAL PRACTICE

This study raised several implications for clinical practice. Our result confirms the importance of introducing person‐cantered care for patients with NS. The care should be organised in a more rigorous way based on the patient's narrative and personal meaning. Second, a health plan is essential to involving patient's and relatives’ abilities and coping strategies. The core problem of the participants was that the disease and its symptoms did not make sense. Thus, it is important to stimulate a continuous learning process with both written and oral information supported by a nurse practitioner or a renal nurse. Further we suggest to implement the five strategies for patient‐centred care proposed by O'Hare (2018), i.e. listening to the patient, making time for the complexity of the patient's situation, being willing to go beyond our job description in order to avoid fragmented care, re‐imagining what it means to provide “good” care and finally to see the value of relationship building. All nursing care is delivered through a caring relationship. This result provides an in‐depth understanding of the illness experience among patients with NS and constitutes a foundation for clinical guidelines on treatment, follow‐up, health promotion as well as renal nursing.

## CONFLICT OF INTEREST

The authors declare no potential conflicts interest with respect to the research, authorship and/or publications of this article.

## AUTHOR CONTRIBUTIONS

AJ, TH and AF: Involved in the study design. AJ: Collected data. AJ and AF: Analyses the data. AJ, TH and AF: Drafted and revised manuscript.
